# A principal-components-based clustering method to identify multiple variants associated with rheumatoid arthritis and arthritis-related autoantibodies

**DOI:** 10.1186/1753-6561-3-s7-s129

**Published:** 2009-12-15

**Authors:** Mary Helen Black, Richard M Watanabe

**Affiliations:** 1 Department of Preventive Medicine, Keck School of Medicine, University of Southern California, 1540 Alcazar Street, CHP 222-V, Los Angeles, California 90089, USA

## Abstract

Multivariate techniques are an important area of investigation for studying contributions of multiple genetic variants to disease onset and pathology. We analyzed the Genetic Analysis Workshop 16 North American Rheumatoid Arthritis Consortium (NARAC) data using a principal-components analysis (PCA) with an orthoblique rotation to identify specific subsets of single-nucleotide polymorphisms (SNP) in the major histocompatibility complex (MHC) region associated with rheumatoid arthritis (RA) and rheumatoid factor IgM (RFUW), and compared this method with a traditional PC approach. Using the orthoblique PC-based clustering method, we identified new clusters of SNPs across the MHC region associated with RA and RFUW, and replicated known SNP cluster associations with RA, such as those in the *HLA-DRB *region.

## Introduction

Testing a candidate gene or region for association with phenotypes typically involves testing multiple single-nucleotide polymorphisms (SNPs). This necessarily introduces the issue of multiple test corrections, which reduce power in order to control the type 1 error rate. Therefore, development of multivariate methods to identify causal loci and to reduce the burden of multiple testing is an area of ongoing investigation for complex diseases. Several multivariate techniques have been used to examine whether multiple SNPs are associated with disease or quantitative traits [1-4]. However, such methods typically suffer from low power under various scenarios or the inability to reduce the large number of SNPs to a smaller subset that may point to a specific location within the region.

Gauderman et al. introduced a principal-components method to assess whether multiple variants within a candidate gene are associated with disease [3]. Principal-components analysis (PCA) is used to derive linear transformations of the original SNP data, in which eigenvectors are chosen to maximize the variance of each PC relative to the overall variation in the region [3]. Each eigenvalue represents the variance of a particular PC, and typically, only a subset of PCs that account for a large proportion of the total variation are chosen for analysis, reducing the number of parameters to be tested. These PCs serve as covariates in an omnibus test of association with disease or trait [3,4].

The PC approach has been shown to have greater power than standard joint SNP or haplotype-based tests to detect association between multiple SNPs and disease, especially when the number of haplotypes is large [3]. However, the coefficients of each eigenvector are derived from pair-wise correlations among the SNPs, and thus lack specific interpretation. Eigenvector loadings of the original variables on a PC do not reflect the true importance of the SNPs to that PC, making the association between multiple PCs and disease outcomes difficult to interpret [4].

We propose a PC-based clustering method as an alternative approach that reduces dimensionality of the data and maintains the power of a PC approach, but allows for unique identification of multiple SNPs in the region being tested. The algorithm uses an orthoblique rotation of PCs on genotype data to form distinct clusters, where each cluster is defined by a specific array of SNPs. A subset of clusters that explains a large proportion of the total locus variation is selected, such that those clusters can be tested for association with disease outcomes.

The PC approach and our proposed oblique PC-based clustering method were applied to the analysis of rheumatoid arthritis (RA) data from Genetic Analysis Workshop 16 (GAW16) (Problem 1). We compare and contrast results from these two approaches and compare findings with previously published results for these data [5-8].

## Methods

### Sample data

Sample data included genome-wide association data from Affymetrix GeneChip 100 k Mapping Array containing 116,204 SNPs for RA and RA-related traits, such as rheumatoid factor IgM (RFUW) and anti-cyclic citrullinated peptide (anti-CCP), on 2,062 North American Rheumatoid Arthritis Consortium (NARAC) subjects. Of the 1,250 SNPs in the major histocompatibility complex (MHC) region on 6p21 spanning 3.2 Mb, we restricted analysis to individuals with RA status and complete genotype data (*n *= 1,187 on 838 SNPs).

### Pre-analysis processing of data

Observed genotype frequencies were assessed for deviation from Hardy-Weinberg equilibrium and allele frequencies estimated using the computer program Haploview (V.4.1). For these analyses, we excluded markers with minor allele frequencies (MAFs) < 0.01, and coded genotypes as 0, 1, or 2 according to the number of minor alleles. Log transforms were applied to quantitative trait data to approximate univariate normality. We performed PCA as described by Gauderman et al. [3]. The subset *s *of PCs used in the analysis was determined by the quantity that accounted for 80% of the total variation.

The PC-based clustering method begins with the total *k *SNPs initially grouped into a single cluster. Standard PCA is performed on the initial cluster, with an orthoblique rotation [9] of the first two PCs (PC_1_, PC_2_). Each SNP is assigned to the rotated component with which it has the higher squared correlation, dividing the initial cluster into two disjointed clusters. PC analysis within newly formed clusters and SNP assignment continue iteratively, assigning SNPs to clusters, and then re-testing each SNP to determine whether assigning it to a different cluster increases the amount of variance explained, with the goal of maximizing the total variance accounted for by the cluster components. For *k *SNPs, we computed *n *clusters:

where C_*n *_is the *n*^th ^cluster score, **c**_**n **_is a vector of standardized cluster coefficients, and **g **is the vector of SNPs [*g*_1_, *g*_2_, ..., *g*_*k*_]. While all SNPs are subdivided into a total of *n *clusters, the number of SNPs within each cluster varies, yielding cluster coefficients equal to zero for SNPs not included in the *n*^th ^cluster. Given the cluster coefficients and individual genotype scores, cluster scores are computed for each individual. For comparison with traditional PC analysis, *n *was determined by the number of clusters that accounted for 80% of the total locus variation.

### Association testing

For disease status, we analyzed PC scores (PC_*s*_) or cluster score (C_*n*_), using the following logistic framework:

For continuous outcomes, we fit a multiple linear regression model with PC scores or cluster scores as covariates. Likelihood-ratio tests were used to contrast the null model (intercept only) to that with either *s *PCs (if traditional PCA) or *n *clusters (if cluster analysis) to assess significance, with *s *or *n *degrees of freedom (df). Given significant association under omnibus test of all PCs or PC-based clusters, 1-df Wald tests were used to test association between RA or RA-related trait with each PC or PC-based cluster conditional upon all PCs or clusters. Because PC and cluster scores are estimated from the correlation structure of the genotype data, it should be noted that *p*-values resulting from any association testing framework may not be completely accurate. A bootstrap or randomization procedure that includes computation of PC scores or cluster components would likely yield more accurate *p*-values. For the purposes of this paper, nominal *p*-values are reported. All analyses were performed using SAS (v.9.1).

## Results

Demographic data for 1,187 subjects (331 male, 856 female) included in this analysis are shown in Table 1. Data analyzed consisted of 515 cases, 672 controls. RA-related traits were measured in cases only. Clinically relevant titres of anti-CCP (≥ 25 U/ml) were found in 98% of cases; for RFUW (≥ 20 IU/ml), it was 95%. Anti-CCP ranged from 20 to 2,554 U/ml, with a mean ± SD of 194.9 ± 234.9 U/ml. RFUW ranged from 9 to 4,225 IU/ml, with mean ± SD 271.0 ± 471.7 IU/ml.

**Table 1:** Subject characteristics

	Cases (*n *= 515)	Controls (*n *= 672)
Sex		
Male *n *(%)	131 (25.4%)	200 (29.8%)
Female *n *(%)	384 (74.6%)	472 (70.2%)
Anti-CCP (units/mL) Mean (SD)	194.9 (234.9)	N/A
RFUW (IU/mL) Mean (SD)	271.1 (471.7)	N/A

Of the 838 SNPs in the MHC region, nine were monomorphic and seven had MAFs < 0.01. PC analysis yielded 53 PCs that accounted for 80% of the variance and were used for association testing. The MHC region was significantly associated with RA status (*p *= 4.8 × 10^-85^) and RFUW (*p *= 0.0159) (Table 2). We found no evidence for association with PCs and anti-CCP (*p *= 0.62). Of the 53 PCs tested for association, 29 individual PCs were significantly associated with RA (*p *≤ 0.05) and 8 were significantly associated with RFUW (*p *≤ 0.05); 5 PCs associated with both RA and RFUW. Among all PCs significantly associated with either RA or RFUW, the eigenvector coefficients ranged from -0.1480 to 0.1453. Therefore, this analysis allows us to conclude that the MHC region is associated with both RA and RFUW, but does not permit us to distinguish the relative contribution of each of the 822 SNPs to this association.

**Table 2:** HLA region association with rheumatoid arthritis and arthritis-related traits

		RA affection status		Anti-CCP		RFUW	
				
Method	df	*χ*^2 ^Statistic^a^	*p*-Value	*χ*^2 ^Statistic^a^	*p*-Value	*χ*^2 ^Statistic^a^	*p*-Value
Traditional PC analysis	53	556.274	4.8 × 10^-85^	49.28	0.6197	77.432	0.0159
PC-based cluster analysis	188	789.007	1.4 × 10^-74^	203.383	0.2098	297.838	5.7 × 10^-7^

^a^*χ*^2 ^statistic based on multi-df likelihood ratio test for number of PCs or clusters included in the full model vs. the null model (intercept only).

Our PC-based clustering algorithm identified 188 clusters that accounted for 80% of the variance and were used to test for association. Cluster size ranged from 1 to 14 SNPs. Using likelihood-ratio tests, the PC-based cluster method also found significant association between the MHC region and RA (*p *= 1.4 × 10^-74^) and RFUW (*p *= 5.7 × 10^-7^) (Table 2). Similar to the PC analysis, the PC-based clustering method showed no evidence for association between the MHC region and anti-CCP (*p *= 0.21). Twenty-four SNP clusters were associated with RA (*p *≤ 0.05) and 36 SNP clusters showed association with RFUW (*p *≤ 0.05); 2 clusters were common to both outcomes.

The two SNP clusters most significantly associated with RA are shown in Table 3. The majority of the SNPs in Cluster 1 are located in the MHC class I region, surrounding *HLA-C *and *HLA-B*. The squared correlation coefficients between each SNP and its assigned cluster (R_O_^2^), shown in Table 3, indicate that most SNPs in Cluster 1 share a high degree of correlation, and relatively low correlation with SNPs in any other cluster (R_N_^2^). Additionally, low values for the ratio of one minus each of these correlations indicates relatively stable SNP-cluster assignment. The SNPs in Cluster 23 primarily span ~295-kb region of MHC class II bounded by *HLA-B *and *HLA-DQA1*. The majority of these SNPs cluster around the *HLA-DRA*, *HLA-DRB5*, and *HLA-DRB1 *loci, are highly correlated with each other, and show stable SNP-cluster assignment.

**Table 3:** Cluster associations with rheumatoid arthritis case-control status

Cluster No.	SNP	Position^a^	MAF	R_O_^2b^	R_N_^2c^	(1-R_O_^2^)/(1-R_N_^2^) Ratio	*p*-Value^d^
1	rs3130544	31,166,319	0.125	0.805	0.682	0.612	0.0002
	rs2442749	31,460,019	0.288	0.346	0.231	0.850	
	rs3099844	31,556,955	0.113	0.897	0.570	0.240	
	rs9267444	31,591,437	0.328	0.332	0.193	0.828	
	rs2734583	31,613,459	0.110	0.917	0.565	0.192	
	rs2857595	31,676,448	0.165	0.648	0.334	0.529	
	rs3131379	31,829,012	0.097	0.906	0.660	0.277	
	rs1270942	32,026,839	0.096	0.885	0.697	0.381	
	rs389884	32,048,876	0.094	0.878	0.707	0.415	
23	rs6910071	32,390,832	0.350	0.675	0.504	0.656	0.0008
	rs3817963	32,476,065	0.402	0.880	0.558	0.271	
	rs3806156	32,481,676	0.453	0.787	0.534	0.458	
	rs3763309	32,483,951	0.358	0.898	0.566	0.235	
	rs2395163	32,495,787	0.355	0.901	0.574	0.232	
	rs2395175	32,513,004	0.312	0.841	0.485	0.308	
	rs2395185	32,541,145	0.444	0.773	0.446	0.411	
	rs2516049	32,678,378	0.429	0.825	0.423	0.303	
	rs660895	32,685,358	0.361	0.832	0.515	0.346	
	rs532098	32,686,030	0.489	0.701	0.312	0.434	

^a^Based on HapMap Data Release 23a (Phase II), NCBI Build 36, dbSNP Build 126

^b^Squared correlation coefficient between a given SNP and its own cluster

^c^The next highest squared correlation coefficient between a given SNP and any other cluster

^d^*p*-Value from 1 df Wald *χ*^2 ^for association with outcome, adjusted for all other clusters in the model

The five SNP clusters most significantly associated with RFUW are shown in Table 4. The majority of the SNPs that comprise Clusters 2, 5, 24, and 183 are located in the MHC class 1 region. Clusters 2, 24, and 183 represent sets of SNPs between *HLA-A *and *HLA-C*, while SNPs in Cluster 5 span a 74-kb region between *HLA-C *and *HLA-B*. Because the SNPs in Cluster 2 reside proximally upstream of *HLA-C *and those in Cluster 5 immediately downstream of the gene, it is likely that both clusters jointly capture the variation in the *HLA-C *locus. The SNPs in Cluster 20 are located in the MHC class II region. Interestingly, three of the nine SNPs in Cluster 20 are located within *HLA-DQB2*, and the remaining six are situated in regions directly flanking *HLA-DQB2*, implying that Cluster 20 may represent the *DQB2 *locus. Also shown in Table 4, all SNPs within each cluster are highly correlated, and each cluster is of relatively stable fit.

**Table 4:** Cluster associations with RFUW

Cluster No.	SNP	Position^a^	MAF	R_O_^2b^	R_N_^2c^	(1-R_O_^2^)/(1-R_N_^2^) Ratio	*p*-Value^d^
2	rs2844670	31,113,705	0.152	0.811	0.488	0.370	0.0004
	rs3130933	31,240,064	0.133	0.945	0.522	0.116	
	rs3094609	31,273,545	0.145	0.954	0.542	0.101	
	rs3130532	31,316,432	0.142	0.956	0.531	0.094	
	rs7382297	31,355,046	0.142	0.953	0.530	0.100	
	rs2905722	31,557,306	0.139	0.747	0.614	0.656	
5	rs11967684	31,307,745	0.353	0.822	0.628	0.477	0.0004
	rs9468925	31,366,816	0.353	0.902	0.464	0.183	
	rs3873379	31,370,148	0.290	0.876	0.478	0.237	
	rs3873380	31,370,417	0.303	0.938	0.499	0.124	
	rs9366778	31,377,152	0.387	0.750	0.517	0.518	
	rs3873386	31,381,724	0.374	0.852	0.514	0.305	
20	rs12177980	32,794,062	0.447	0.888	0.443	0.202	0.0003
	rs9461799	32,797,507	0.447	0.888	0.443	0.202	
	rs13199787	32,813,254	0.449	0.882	0.436	0.209	
	rs10807113	32,830,164	0.462	0.890	0.471	0.209	
	rs7756516	32,831,895	0.462	0.890	0.471	0.209	
	rs2301271	32,833,171	0.413	0.856	0.427	0.251	
	rs7453920	32,837,990	0.413	0.856	0.425	0.250	
	rs6903130	32,840,188	0.495	0.820	0.369	0.286	
	rs6901084	32,844,914	0.469	0.890	0.432	0.194	
24	rs9468841	30,933,266	0.076	0.570	0.146	0.504	0.0007
	rs7756521	30,956,232	0.178	0.888	0.360	0.175	
	rs3873334	31,004,126	0.167	0.929	0.336	0.107	
	rs12697941	31,012,693	0.152	0.928	0.302	0.104	
	rs3757340	31,029,861	0.270	0.554	0.307	0.644	
183	rs3868542	31,253,818	0.370	0.772	0.366	0.360	0.0001
	rs887464	31,253,899	0.431	0.709	0.276	0.402	
	rs4122189	31,275,906	0.233	0.795	0.556	0.462	

^a^Based on HapMap Data Release 23a (Phase II), NCBI Build 36, dbSNP Build 126

^b^Squared correlation coefficient between a given SNP and its own cluster

^c^The next highest squared correlation coefficient between a given SNP and any other cluster

^d^*p*-Value from 1 df Wald *χ*^2 ^for association with outcome, adjusted for all other clusters in the model

## Discussion

Traditionally, investigators examining gene regions or specific candidate genes might genotype hundreds of SNPs, possibly perform tag SNP selection, and test each SNP for association with disease or disease-related traits. Unfortunately, this approach necessitates multiple test correction, resulting in a significant reduction in power. PC analysis has been suggested as an exploratory approach that parses the information contained in a large number of correlated SNPs into a smaller number of orthogonal PCs that can be analyzed for association instead of individual SNPs [3,4]. A significant omnibus test of PCs indicates statistical association between a given region, as represented by the SNPs genotyped, and disease outcomes. However, PCA cannot be used to identify the specific SNPs contributing to the association, and therefore still requires testing of individual variants, to isolate the specific SNP(s) contributing to the association. We introduce a PC-based clustering method that retains many of the favorable attributes of PC regression, but allows for identification of the subset of SNPs contributing to the evidence for association, which reduces the multiple testing burden. We compared the traditional PC approach to the PC-clustering method using the NARAC data, and demonstrate that PC-clustering identifies variants in the 3.2-Mb MHC region contributing to RA risk and variation in RA-related traits.

While traditional PC analysis makes it possible to analyze only the subset of PCs that represent most of the variation in a candidate region, PCs still represent linear combinations of all SNPs in the data set, which makes interpretation of significant PCs difficult. Upon inspection of the 29 PCs from the full model found to be significantly associated with RA status, we found the 822 eigenvector loadings on these PCs to range from -0.148 to 0.145, with most hovering close to 0. Thus, we were only able to infer from PC analysis that variation in the MHC region, as represented by these 822 SNPs, is strongly associated with RA risk. Additional interpretation of the specific SNP(s) driving significant associations between PCs and phenotypes can only be achieved by testing all 822 SNPs individually for association. In contrast, the PC-based clustering algorithm we employed reduced 822 SNPs to 188 discernable SNP clusters that also accounted for 80% of the regional variation. The clusters, which are subsets of the 822 SNPs analyzed, allow unique identification of those SNPs that may contribute to the evidence for association. For example, of the 24 SNP clusters associated with RA status, Cluster 1 and Cluster 23 were found to be the most significant. Cluster 1 represents a distinct set of SNPs covering ~883 kb of the 3.2-Mb region examined, while Cluster 24 covers a non-overlapping region of ~295 kb. While Cluster 1 represents SNPs flanking *HLA-C *and *HLA-B*, Cluster 23 comprises SNPs surrounding the *HLA-DRA*, *HLA-DRB5*, and *HLA-DRB1 *loci. In fact, rs3099844 and rs2857595 found in Cluster 1 were previously identified by Lee et al. [5] as belonging to a haplotype associated with anti-CCP positive RA, which 98% of cases in the present study were. Additionally, rs2395175 in Cluster 23 ranked among the top ten SNPs for association with RA in a recent genome-wide association study by Plenge et al. [8].

The clustering algorithm also identified 36 SNP clusters found to be associated with variation in RFUW among RA cases. The most significant associations included Clusters 2, 5, 20, 24, and 183. Clusters 2, 5, 24, and 183 are composed of SNPs located in the chromosomal region between *HLA-A *and *HLA-C*, with Clusters 2 and 5 capturing the specific variation in and around *HLA-C*. Interestingly, Yen et al. demonstrated that *HLA-C *alleles may modulate the pattern of RA progression [10]. Moreover, Lee et al. found rs887464 in Cluster 183 to be associated with RA affection [5]. Cluster 20, composed of nine SNPs, represents variants located within and proximal to *HLA-DQB2*. Previous examination of genes in the MHC class II region, conditional on the *HLA-DRB *loci, has shown the *HLA-DQB2 *locus to have a vital role in RA [11,12]. As RA is heterogeneous in terms of the progression of joint destruction [13], further examination of the SNPs in these clusters may provide information regarding genetic determinants of RA progression or symptom severity.

While our PC-based clustering method offers the interpretability a traditional PC approach lacks, there are other issues to be considered. First, we required more clusters than PCs to satisfy the 80% explained-variance threshold, which increased the degrees of freedom utilized for the omnibus test of association. The additional degrees of freedom usually results in reduced power to detect global association compared to the traditional PC approach. This may be due to the fact that while PCs are orthogonal, or independent, cluster components formed by the clustering algorithm are oblique. At each iteration, PC_1 _and PC_2 _are computed from a distinct set of SNPs that have been assigned to a given cluster, such that the first PC of one cluster may be correlated with the first PC of another cluster. Thus, although each SNP is assigned to the cluster with which it has the highest squared correlation, all SNPs share some degree of correlation with the other clusters they were not assigned to. This underlying correlation among clusters may be indicative of the correlation pattern among SNPs, although not necessarily haplotype blocks, and thus better reflect the true relationship of the variants within the MHC candidate region, but may also result in slightly reduced power to detect association.

## Conclusion

Both traditional PC and PC-based clustering methods indicate the MHC gene region is significantly associated with RA and RFUW. However, traditional PCA is unable to highlight which SNPs contributed to this association. In contrast, the PC-based clustering method maintains many of the virtues of the traditional PC approach, but has the advantage of isolating the SNP(s) contributing to evidence for association. Therefore, the PC-based clustering method may be a better approach to testing multiple variant associations with phenotypes of interest.

## List of abbreviations used

Anti-CCP: Anti-cyclic citrullinated peptide; GAW16: Genetic Analysis Workshop 16; MAF: Minor allele frequency; MHC: Major histocompatability complex; NARAC: North American Rheumatoid Arthritis Consortium; PCA: Principal-components analysis; RA: Rheumatoid arthritis; RFUW: Rheumatoid factor IgM; SNP: Single-nucleotide polymorphism.

## Competing interests

The authors declare that they have no competing interests.

## Authors' contributions

MHB conceived of the study, analyzed the data, and drafted the manuscript. RMW advised on the manuscript. Both authors read and approved the final manuscript.
